# Force analysis of slope reinforced by Frame-Anchor-Support-Structure based on improved horizontal slice method

**DOI:** 10.1371/journal.pone.0317361

**Published:** 2025-02-07

**Authors:** Guang-Wen Fang, Fei Guo, An-Ping Huang

**Affiliations:** School of Environment and Urban Construction, Lanzhou City University, Lanzhou, China; Guizhou University, CHINA

## Abstract

As a flexible support structure commonly used in slope stabilization and management, the Frame-Anchor-Support-Structure (FASS) is of great significance in studying the actual stress situation to ensure the safety of slopes. In this paper, based on the traditional horizontal slice method, utilizing the principle that the vertical slice method and the horizontal slice method share the same sliding surface, the inter-strip force between soil slices, the effect of the FASS, the effect of the slope-top uniform load, and the relationship between the vertical slice method and the horizontal slice method of the force conversion is considered at the same time. The improved horizontal slice method for the slope reinforced by the FASS is also proposed, and the force calculation formula for the slope reinforced by FASS is derived. By comparing and analyzing with the overall sliding wedge method, the reasonableness and accuracy of the calculation method of this paper are verified, and the variety rule of force on the slope reinforced by FASS under different loads is obtained. The results show that the combined force of lateral earth pressures on slopes calculated in this paper is smaller than that of the overall sliding wedge method, but the difference between the two is not significant (within 9% difference). The earth pressure strength of the slope, the shear stress of FASS on the sliding soil, and the vertical force strength and the shear stress of the stabilized soil on the sliding soil all show a distribution trend of small at both ends and large in the middle (about 1/3 of the position from the bottom of the slope). Moreover, the trend of the above forces does not vary with the slope-top uniform loads. The study results are informative for calculating and analyzing the forces on the slope reinforced by FASS.

## 1. Introduction

The Frame-Anchor-Support-Structure (FASS) has been widely used in slope support engineering in recent years due to its strong deformation control ability, good support effect, easy construction, and other advantages [1–6]. The earth pressure is the lateral pressure on the supporting structure due to the self-weight of soil and external loads. The earth pressure changes with the displacement of the supporting structure and exhibits nonlinear characteristics. The study of the magnitude and variation of earth pressure can accurately determine the stability of slopes. The FASS bears the earth pressure generated by the slope soil through the frame lattice structure, and uses the anchors connected with the frame lattice structure to transfer the force to the anchored section located in the stabilized soil layer, to achieve the purpose of reinforcing the slope and controlling the deformation. Analyzing the working mechanism of the FASS, it can be found that the external force borne in the structure is mainly the earth pressure of the slope, i.e., the earth pressure of the slope acts on the structure, and the structure starts to work normally. Therefore, the key to study the actual force system of FASS lies in the establishment of a computational analysis model that is more in line with the earth pressure of the slope reinforced by FASS, and based on this computational analysis model, the relevant force analysis of the slope reinforced by FASS is carried out.

Currently, most of the classical Rankine [7] and Coulomb [8] earth pressure calculation theories are used for calculating slope earth pressure. In Rankine’s earth pressure theory, the retaining wall is assumed to be smooth, whereas Coulomb’s theory considers the soil-wall friction. The earth pressure derived from the classical earth pressure calculation theory shows a linear distribution along the slope, but a large number of experimental studies [9–10] and numerical simulation results [11–12] have confirmed the nonlinear distribution characteristics of earth pressure [13–14]. In order to characterize the distribution of nonlinear earth pressure, many scholars have carried out relevant research work from the aspects of static state, seismic action and rainfall infiltration, respectively [15–16]. Fathipour et al. [17] comprehensively evaluated the effect of transient flow on active and passive lateral nonlinear earth pressures in variably saturated backfill by using the lower bound theorem of second-order cone-planning finite element limit analysis. Nguyen [18–20] derived an exact solution of active and passive nonlinear earth pressures for retaining walls, considering multiple influences based on the static allowable stress field. In response to the current situation where linear slip surfaces are mostly used in analyzing earth pressure, Garzón and Hanna [21] give an alternative theoretical explicit nonlinear solution for determining the active and passive coefficients of lateral earth pressures and the nonlinear slip-failure surface geometry through a rigorous mathematical analysis based on the variational limit-equilibrium method, which increases the accuracy of nonlinear earth pressure calculations. Leshchinsky and Zhu [22] proposed a method for solving lateral nonlinearly distributed earth pressures on unstable slopes with slope angles less than 90 degrees. On the other hand, Deng et al. [23–24] analyzed and researched the calculation and variety rule of earth pressure of retaining structures under unsaturated soil, and derived a nonlinear earth pressure calculation method for unsaturated soil. According to the phenomenon of only considering soil arching effects without considering interlayer shear in the calculation of earth pressure on vertical rigid retaining walls, Li et al. [25] proposed a model for calculating the earth pressure considering the interlayer shear, wall displacement, and soil arching effect based on the horizontal slice method. In addition to studying the nonlinear distribution of earth pressure, scholars have begun to study the relationship between nonlinear earth pressure and displacement. Liang et al. [12] proposed a simplified method based on hyperbolic modeling for calculating the magnitude and distribution of lateral earth pressures corresponding to different displacements in the active state of a retaining wall.

In addition, in order to further make up for the shortcomings of the Coulomb earth pressure analysis method, which is difficult to reasonably and accurately determine the size and distribution of earth pressure, and to seek an easy-to-operate method in practice that can determine the distribution pattern of earth pressure, a large number of scholars have introduced the horizontal slice method based on the theory of limit equilibrium [26–30]. Deng and Li [31] established the assumption conditions of inter-strip force between soil slices corresponding to the horizontal slice method and vertical slice method by studying the relationship of between the horizontal slice method and the vertical slice method, which thoroughly considered the inter-strip force between soil slices. The research object of this method is unreinforced slopes. Chandaluri et al. [32] analyzed the effect of various parameters on the seismic stability of reinforced soil walls using the horizontal slice method. However, when the method analyzes the force of horizontal soil slice, the horizontal force between horizontal soil slices is offset by overall summation, and the horizontal force between horizontal soil slices is not analyzed. and the force of the retaining wall surface on the surface of the slope is not analyzed. And the force of the retaining wall on the surface of the slope was not analyzed. Farshidfar et al. [33] performed a pseudo-static seismic analysis of reinforced soil slope with an arbitrary slip surface considering the top of slope loading using the horizontal slice method. Although the method considers the external load and the force between the horizontal soil slices, the consideration of the vertical force and the horizontal force of the horizontal soil slices is also simplified, and it does not take into account the forces of the structure of the reinforced soil on the surface of the slope. Agarwal and Pain [34] used the rigorous (5N-1) formulation of the horizontal slicing method to analyze the forces on the geosynthetic reinforced slopes. Although the method considers the interaction between horizontal soil slices, it only assumes that there is a simple functional relationship between the vertical force and the horizontal force of the horizontal soil slice, and does not explore the interaction between horizontal soil slices in depth. And external loading effects were also not considered. Yazdandoust [35] has established the equivalent seismic coefficients required for slip surfaces similar to those observed in the shaking table tests, using the limit equilibrium horizontal slice method and based on the results of shaker tests. However, the method does not take into account the horizontal forces between horizontal soil slices. To address this problem, Yazdandoust et al. [36] applied the Morgenstern and Price equation based on the above to take into account the horizontal forces between horizontal soil slices, and further accurately calculated the equivalent seismic coefficients. And, both of these papers do not consider the forces acting on the surface of the slope by reinforced structure. Based on a large number of previous modeling tests and field monitoring of earth pressure data, Chen and Xiao [37] introduced the value of soil shear strength exertion, and established a method for calculating the earth pressure of rigid retaining walls with full consideration of the inter-strip force between soil slices through the horizontal slice limit equilibrium method. However, the earth pressure calculation analysis of slope reinforced by FASS is less frequently performed by the horizontal slice method. Most existing studies are also based on the Coulomb earth pressure theory calculation and simplify the distribution of lateral earth pressure on slopes, which in turn gives the lateral earth pressure of the slope reinforced by FASS [38–39]. And, this earth pressure calculation method does not consider the actual force characteristics of the slope reinforced by FASS and the actual factors affecting the distribution of lateral earth pressure on slopes.

Analyzing the above-existing research status on lateral earth pressure on slopes, the following primary research deficiencies can be found: (1) Most lateral earth pressure studies on slopes require that the back of the slope support structure wall be vertical or that the slope inclination behind the wall be >90°, but there are fewer studies related to earth pressure for slope inclinations <90°; (2) The potential sliding surface of slopes is usually assumed to be folded or straight not circular or log spiral, which deviates significantly from the reality; (3) Most of the lateral earth pressure solutions for slopes require the lateral earth pressure coefficient to be solved first, and the accuracy of the calculations needs to be improved; (4) The main object of study is rigid retaining walls, and less research has been done on flexible support structures; (5) For the calculation of lateral earth pressure on slopes with flexible support structure and slope inclination <90°, the effect of inter-strip force between soil slices and the friction force of the support structure on the slope surface are less considered, and the calculated slope earth pressures do not reflect the true forces on the slope. Therefore, the paper addresses the above problems in the calculation of lateral earth pressure on slopes, and takes the typical flexible support structure—FASS as the research object. In this paper, based on the traditional horizontal slice method, the circular sliding surface method is used to determine the sliding surface for slopes with an inclination of <90°, and utilizing the principle that the vertical slice method and the horizontal slice method share the same sliding surface, the force conversion relationship between the vertical slice method and the horizontal slice method is established, and the improved horizontal slice method for slope reinforced by the FASS is proposed. Compared with the traditional method, this method can consider the inter-strip force between soil slices, the effect of the FASS, the effect of the slope-top uniform load, the relationship between the vertical slice method and the horizontal slice method of the force conversion at the same time, which makes up for the shortcomings of the traditional method, reflects the true forces on the slope, and improves the accuracy of the calculation and analysis of the relevant forces of slope reinforced by the FASS.

## 2. Basic principle of the improved horizontal slice method for slope reinforced by the FASS

This method proposes an improved horizontal slice method for slope reinforced by the FASS based on the traditional horizontal slice method by utilizing the principle that the vertical slice method and the horizontal slice method share the same sliding surface. Furthermore the method takes into account a variety of influencing factors, and it makes up for the shortcomings of the traditional horizontal slice method mentioned above.

**Fig 1:**
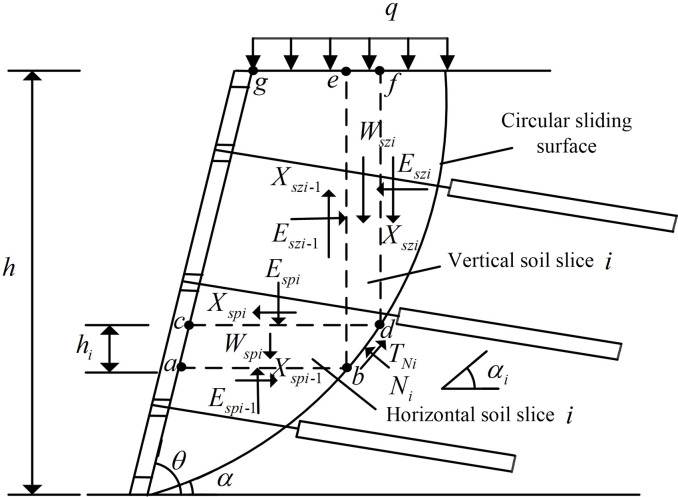
Analytical model for slope reinforced by FASS.

The analysis model of slope reinforced by FASS is established, as shown in Fig 1 (the calculated width is taken as unit width): abdc represents a horizontal soil slice *i*; ebdf represents a vertical soil slice *i*; *q* represents the vertical uniform load acting at the top of the slope, and $X_{spi}$ and $X_{spi-1}$ represent the inter-strip shear forces at the top and bottom of the horizontal soil slice, respectively; $E_{spi}$ and $E_{spi-1}$ represent the inter-strip normal forces on the upper and lower bottom surfaces of the horizontal soil slice *i*, respectively; $X_{szi}$ and $X_{szi-1}$ represent the inter-strip shear forces between the left and right sides of the vertical soil slice *i*, respectively; $E_{szi}$ and $E_{szi-1}$ represent the inter-strip normal forces between the left and right sides of the vertical soil slice *i*, respectively; $N_{i}$ represents the vertical force on the soil slice from the sliding bottom surface common to the horizontal soil slice *i* and vertical soil slice *i*; $T_{Ni}$ represents the shear force on the soil slice from the sliding bottom surface common to the horizontal soil slice *i* and vertical soil slice *i*; $W_{spi}$ and $W_{szi}$ represent the gravity of horizontal soil slice *i* and vertical soil slice *i*, respectively.

**Fig 2:**
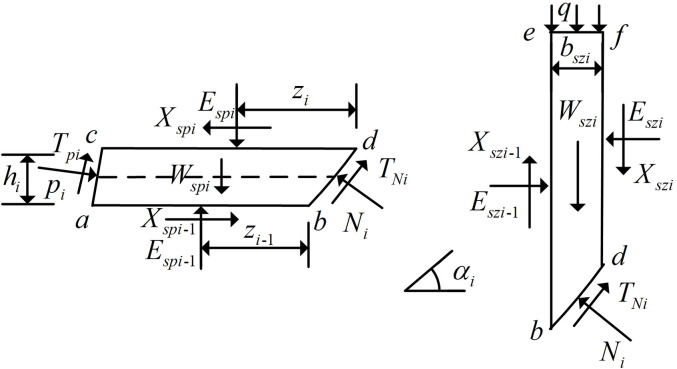
Horizontal soil slice and Vertical soil slice.

**Fig 3:**
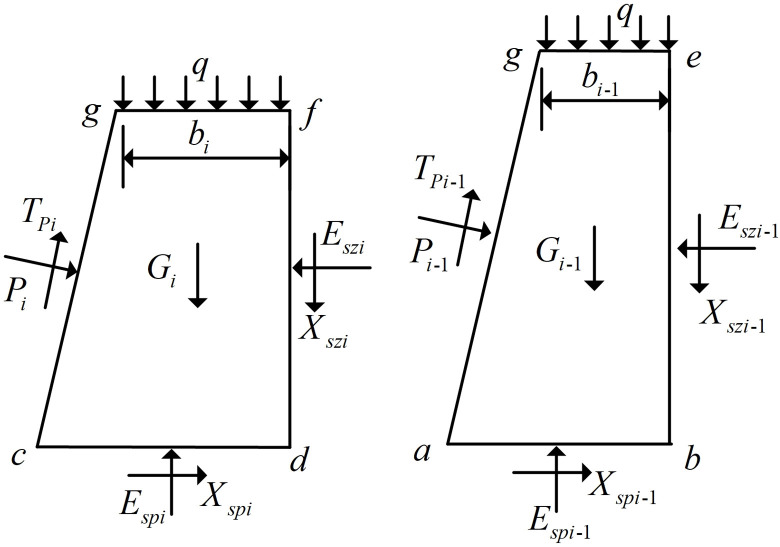
Block corresponding to Horizontal soil slice.

In order to establish the force relationship between the horizontal soil slice *i* and vertical soil slice *i*, abdc, ebdf, cdfg and abeg are taken as separations for force analysis. Separations abdc and ebdf are shown in Fig 2, and separations cdfg and abeg are shown in Fig 3, where the block corresponding to the upper top face of the horizontal soil slice *i* is cdfg, and the block corresponding to the lower bottom face of the horizontal soil slice *i* is abeg.

As shown in Fig 2, $\alpha_{i}$ in the figure indicates the angle between the corresponding circular slip fracture surface of the soil slice *i* and the horizontal plane; $z_{i}$ and $z_{i-1}$ respectively represent the distances of the upper bottom inter-strip normal force and the lower bottom inter-strip normal force from the circular slip fracture surface of the horizontal soil slice *i*; $p_{i}$ represents the vertical force of the FASS on the horizontal soil slice *i*; $T_{pi}$ represents the shear force of the FASS on the horizontal soil slice *i*; $h_{i}$ represents the height of the horizontal soil slice *i*; $\varphi_{i}$ represents the angle of internal friction of the soil slice *i*; $b_{szi}$ represents the width of the vertical soil slice *i*.

As shown in Fig 3, $G_{i}$ represents the gravity on the block cdfg corresponding to the top surface of the horizontal soil slice *i*; $G_{i-1}$ represents the gravity on the block abeg corresponding to the bottom surface of the horizontal soil slice *i*; $P_{i}$ represents the vertical force exerted by the FASS on the block cdfg; $T_{pi}$ represents the shear force exerted by the FASS on the block cdfg; $P_{i-1}$ represents the vertical force exerted by the FASS on the block abeg; $T_{pi-1}$ represents the shear force exerted by the FASS on the block abeg; $b_{i}$ and $b_{i-1}$ represent the top widths of block cdfg and block abeg, respectively, and the top width is 0 when the horizontal soil slice corresponding to the block is triangular.

## 3. Force analysis of the improved horizontal slice method for slope reinforced by the FASS

To analyse the force on the horizontal soil slice *i*, the vertical force $p_{i}$ acting on the soil slice by the FASS is decomposed into the horizontal component $p_{i}^{h}$ and the vertical component $p_{i}^{v}$; The shear force $T_{pi}$ acting on the soil slice by the FASS is decomposed into the horizontal component $T_{pi}^{h}$ and the vertical component $T_{pi}^{v}$; The shear force $T_{Ni}$ acting on the soil slice from the sliding surface is decomposed into the horizontal component $T_{Ni}^{h}$ and the vertical component $T_{Ni}^{v}$; The vertical force $N_{i}$ acting on the soil slice from the sliding surface is decomposed into the horizontal component $N_{i}^{h}$ and the vertical component $N_{i}^{v}$, i.e.,


$$\{\begin{array}{l} p_{i}^{h}=p_{i}\sin\theta \;\left(\to \right)\\ p_{i}^{v}=p_{i}\cos\theta \;\left(↓\right) \end{array}\; \{\begin{array}{l} T_{pi}^{h}=T_{pi}\cos\theta \;\left(\to \right)\\ T_{pi}^{v}=T_{pi}\sin\theta \;\left(↑\right) \end{array}$$ (1)

$$\{\begin{array}{l} N_{i}^{h}=N_{i}\sin\alpha_{i}\;\left(\leftarrow \right)\\ N_{i}^{v}=N_{i}\cos\alpha_{i}\;\left(↑\right) \end{array}\; \{\begin{array}{l} T_{Ni}^{h}=T_{Ni}\cos\alpha_{i}\;\left(\to \right)\\ T_{Ni}^{v}=T_{Ni}\sin\alpha_{i}\;\left(↑\right) \end{array}$$ (2)

where the arrows in parentheses indicate the direction of action of the force components.

According to the Moore Cullen criterion, the shear force $T_{pi}$ acting on the horizontal soil slice *i* by the FASS and the shear force $T_{Ni}$ acting on the horizontal soil slice *i* by the sliding surface can be expressed as follows:


$$\{\begin{array}{l} T_{pi}=p_{i}\tan\delta_{i}+c_{wi}\frac{h_{i}}{\sin\theta }\\ T_{Ni}=N_{i}\tan\varphi_{i}+c_{i}\frac{h_{i}}{\sin\alpha_{i}} \end{array}$$ (3)

where $\delta_{i}$ is the friction angle between the wall and soil at the location of the horizontal soil slice *i*, $c_{wi}$ is the cohesion between the wall and soil at the location of the horizontal soil slice *i*, $\varphi_{i}$ is the internal friction angle of the soil at the location of the horizontal soil slice *i* corresponding to the sliding surface, and $c_{i}$ is the cohesion of the soil at the location of the horizontal soil slice *i* corresponding to the sliding surface.

The force equilibrium analysis of the horizontal soil slice *i* is carried out and its static equilibrium equation in the vertical direction is given as:


$$W_{spi}+E_{spi}+p_{i}^{v}=N_{i}^{v}+T_{pi}^{v}+E_{spi\text{-}1}+T_{Ni}^{v}$$ (4)

The static equilibrium equation for the horizontal soil slice *i* in the horizontal direction is:


$$X_{spi}+N_{i}^{h}=X_{spi\text{-}1}+T_{pi}^{h}+p_{i}^{h}+T_{Ni}^{h}$$ (5)

The force analysis of block cdfg and block abeg shown in Fig 3 is carried out, and decompose the forces on the blocks into horizontal forces (superscripted as *h*) and vertical forces (superscripted as *v*).


$$\{\begin{array}{l} P_{i}^{h}=P_{i}\sin\theta \;\left(\to \right)\\ P_{i}^{v}=P_{i}\cos\theta \;\left(↓\right) \end{array}\; \{\begin{array}{l} P_{i-1}^{h}=P_{i-1}\sin\theta \;\left(\to \right)\\ P_{i-1}^{v}=P_{i-1}\cos\theta \;\left(↓\right) \end{array}$$ (6)

$$\{\begin{array}{l} T_{Pi}^{h}=T_{Pi}\cos\theta \;\left(\to \right)\\ T_{Pi}^{v}=T_{Pi}\sin\theta \;\left(↑\right) \end{array}\; \{\begin{array}{l} T_{Pi-1}^{h}=T_{Pi-1}\cos\theta \;\left(\to \right)\\ T_{Pi-1}^{v}=T_{Pi-1}\sin\theta \;\left(↑\right) \end{array}$$ (7)

The force equilibrium analyses of block cdfg and block abeg are carried out and their static equilibrium equations in the vertical direction are respectively:


$$\{\begin{array}{l} qb_{i}+P_{i}^{v}+G_{i}+X_{szi}=E_{sqi}+T_{Pi}^{v}\\ qb_{i-1}+P_{i-1}^{v}+G_{i-1}+X_{szi-1}=E_{sqi-1}+T_{Pi-1}^{v} \end{array}$$ (8)

The equations of static equilibrium between block cdfg and block abeg in the horizontal direction are:


$$\{\begin{array}{l} P_{i}^{h}+X_{spi}+T_{Pi}^{h}=E_{szi}\\ P_{i-1}^{h}+X_{spi-1}+T_{Pi-1}^{h}=E_{szi-1} \end{array}$$ (9)

The expressions for the calculation of the vertical force $p_{i}$ acting on the soil slice by the FASS and the vertical force $N_{i}$ acting on the soil slice by the sliding surface can be obtained by associating Eq (1) to Eq (5), respectively, as follows:


$$\{\begin{array}{l} p_{i}=\frac{B\left[\left(X_{spi-1}-X_{spi}\right)+c_{wi}h_{i}\cdot \cot\theta +c_{i}h_{i}\cdot \cot\alpha_{i}\right]+D\left[\left(E_{spi-1}-E_{spi}\right)-W_{spi}+c_{wi}h_{i}+c_{i}h_{i}\right]}{AD-BC}\\ N_{i}=\frac{A\left[\left(X_{spi-1}-X_{spi}\right)+c_{wi}h_{i}\cdot \cot\theta +c_{i}h_{i}\cdot \cot\alpha_{i}\right]+C\left[\left(E_{spi-1}-E_{spi}\right)-W_{spi}+c_{wi}h_{i}+c_{i}h_{i}\right]}{AD-BC} \end{array}$$ (10)

where


$$\{\begin{array}{l} A=\cos\theta -\tan\delta_{i}\cdot \sin\theta \\ B=\cos\alpha_{i}+\tan\varphi_{i}\cdot \sin\alpha_{i}\\ C=\sin\theta +\tan\delta_{i}\cdot \cos\theta \\ D=\sin\alpha_{i}-\tan\varphi_{i}\cdot \cos\alpha_{i} \end{array}$$ (11)

According to Eq (10), in order to solve for the vertical force $p_{i}$ on the soil slice by the FASS and the vertical force $N_{i}$ on the soil slice by the sliding surface, it is necessary to determine $\left(X_{spi-1}-X_{spi}\right)$ and $\left(E_{spi-1}-E_{spi}\right)$ as well. According to Eqs (6)–(9),


$$\{\begin{array}{l} X_{spi-1}-X_{spi}=\left(E_{szi-1}-E_{szi}\right)-\left(P_{i-1}^{h}-P_{i}^{h}\right)-\left(T_{Pi-1}^{h}-T_{Pi}^{h}\right)\\ E_{spi-1}-E_{spi}=q\left(b_{i-1}-b_{i}\right)+\left(G_{i-1}-G_{i}\right)+\left(X_{szi-1}-X_{szi}\right)+\left(P_{i-1}^{v}-P_{i}^{v}\right)-\left(T_{Pi-1}^{v}-T_{Pi}^{v}\right) \end{array}$$ (12)

where


$$\{\begin{array}{l} P_{i-1}^{h}-P_{i}^{h}=p_{i}^{h}=p_{i}\sin\theta \\ P_{i-1}^{v}-P_{i}^{v}=p_{i}^{v}=p_{i}\cos\theta \end{array}\; \{\begin{array}{l} T_{Pi-1}^{h}-T_{Pi}^{h}=T_{pi}^{h}=p_{i}\cos\theta \cdot \tan\delta_{i}+c_{wi}h_{i}\cdot \cot\theta \\ T_{Pi-1}^{v}-T_{Pi}^{v}=T_{pi}^{v}=p_{i}\sin\theta \cdot \tan\delta_{i}+c_{wi}h_{i} \end{array}$$ (13)

Substituting Eq (13) into Eq (12) yields that


$$\{\begin{array}{l} X_{spi-1}-X_{spi}=\left(E_{szi-1}-E_{szi}\right)-p_{i}\sin\theta -\left(p_{i}\cos\theta \cdot \tan\delta_{i}+c_{wi}h_{i}\cdot \cot\theta \right)\\ E_{spi-1}-E_{spi}=q\left(b_{i-1}-b_{i}\right)+\left(G_{i-1}-G_{i}\right)+\left(X_{szi-1}-X_{szi}\right)+p_{i}\cos\theta -\left(p_{i}\sin\theta \cdot \tan\delta_{i}+c_{wi}h_{i}\right) \end{array}$$ (14)

Substituting Eq (14) into Eq (10) yields an analytical expression for the vertical force $p_{i}$ acting on the soil slice by the FASS and the vertical force $N_{i}$ acting on the soil slice by the sliding surface, as shown in Eq (15):


$$\{\begin{array}{l} p_{i}=\frac{B\left\{\left[\left(E_{szi-1}-E_{szi}\right)-p_{i}\sin\theta -\left(p_{i}\cos\theta \cdot \tan\delta_{i}+c_{wi}h_{i}\cdot \cot\theta \right)\right]+c_{wi}h_{i}\cdot \cot\theta +c_{i}h_{i}\cdot \cot\alpha_{i}\right\}}{AD-BC}\\ \text{ +}\frac{D\left\{\left[q\left(b_{i-1}-b_{i}\right)+\left(G_{i-1}-G_{i}\right)+\left(X_{szi-1}-X_{szi}\right)+p_{i}\cos\theta -\left(p_{i}\sin\theta \cdot \tan\delta_{i}+c_{wi}h_{i}\right)\right]-W_{spi}+c_{wi}h_{i}+c_{i}h\right\}}{AD-BC}\\ N_{i}=\frac{A\left\{\left[\left(E_{szi-1}-E_{szi}\right)-p_{i}\sin\theta -\left(p_{i}\cos\theta \cdot \tan\delta_{i}+c_{wi}h_{i}\cdot \cot\theta \right)\right]+c_{wi}h_{i}\cdot \cot\theta +c_{i}h_{i}\cdot \cot\alpha_{i}\right\}}{AD-BC}\\ \text{ +}\frac{C\left\{\left[q\left(b_{i-1}-b_{i}\right)+\left(G_{i-1}-G_{i}\right)+\left(X_{szi-1}-X_{szi}\right)+p_{i}\cos\theta -\left(p_{i}\sin\theta \cdot \tan\delta_{i}+c_{wi}h_{i}\right)\right]-W_{spi}+c_{wi}h_{i}+c_{i}h\right\}}{AD-BC} \end{array}$$ (15)

In order to make the calculation of Eq (15) more simple, and taking into account that the soil in the sliding area is whole without mutual misalignment between soil slices, the following assumptions are made in this paper in calculating the lateral earth pressure of the slope reinforced by the FASS (as shown in Eq (16)):

(1) Approximate the difference in inter-strip shear force between vertical soil slices *i* as 0;
(2) Approximate the difference in inter-strip shear force for horizontal soil slices *i* as 0.

$$\{\begin{array}{l} \Delta X_{szi}=X_{szi}-X_{szi-1}=X_{szi-1}-X_{szi}=0\\ \Delta X_{spi}=X_{spi}-X_{spi-1}=X_{spi-1}-X_{spi}=0 \end{array}$$ (16)

Associating Eq (10), Eq (14), Eq (15) and Eq (16), the simplified analytical expressions of the vertical force $p_{i}$ acting on the soil slice by the FASS and the vertical force $N_{i}$ acting on the soil slice by the sliding surface can be obtained, as shown in Eq (17).


$$\{\begin{array}{l} p_{i}=\frac{D\left[q\left(b_{i}-b_{i-1}\right)+\left(G_{i}-G_{i-1}\right)+W_{spi}-c_{i}h_{i}\right]-B\left(c_{wi}h_{i}\cdot \cot\theta +c_{i}h_{i}\cdot \cot\alpha_{i}\right)}{BC}\\ N_{i}=\frac{q\left(b_{i}-b_{i-1}\right)+\left(G_{i}-G_{i-1}\right)+W_{spi}-c_{i}h_{i}}{B} \end{array}$$ (17)

The analytical expressions for the shear force $T_{pi}$ acting on the horizontal soil slice by the FASS and the shear force $T_{Ni}$ acting on the horizontal soil slice by the sliding surface can be obtained by associating Eq (3) with Eq (17), as shown in Eq (18).


$$\{\begin{array}{l} T_{pi}=\left\{\frac{D\left[q\left(b_{i}-b_{i-1}\right)+\left(G_{i}-G_{i-1}\right)+W_{spi}-c_{i}h_{i}\right]-B\left(c_{wi}h_{i}\cdot \cot\theta +c_{i}h_{i}\cdot \cot\alpha_{i}\right)}{BC}\right\}\cdot \tan\delta_{i}+c_{wi}\cdot \frac{h_{i}}{\sin\theta }\\ T_{Ni}=\left[\frac{q\left(b_{i}-b_{i-1}\right)+\left(G_{i}-G_{i-1}\right)+W_{spi}-c_{i}h_{i}}{B}\right]\cdot \tan\varphi_{i}+c_{i}\cdot \frac{h_{i}}{\sin\alpha_{i}} \end{array}$$ (18)

Let the relationship between the vertical force $p_{i}$ and the vertical force strength $\sigma_{pi}$ acting on the horizontal soil slice *i* by the FASS, the shear force $T_{pi}$ and the shear stress $\tau_{pi}$ acting on the horizontal soil slice *i* by the FASS, the vertical force $N_{i}$ and the vertical force strength $\sigma_{Ni}$ acting on the horizontal soil slice *i* by the sliding surface, and the shear force $T_{Ni}$ and the shear stress $\tau_{Ni}$ acting on the horizontal soil slice *i* by the sliding surface be shown in Eq (19).


$$\{\begin{array}{l} \sigma_{pi}=\frac{p_{i}\cdot \sin\theta }{h_{i}}\\ \tau_{pi}=\frac{T_{pi}\cdot \sin\theta }{h_{i}} \end{array}\; \{\begin{array}{l} \sigma_{Ni}=\frac{N_{i}\cdot \sin\alpha_{i}}{h_{i}}\\ \tau_{Ni}=\frac{T_{Ni}\cdot \sin\alpha_{i}}{h_{i}} \end{array}$$ (19)

The analytical expressions for the vertical force $p_{i}$ and the vertical force strength $\sigma_{pi}$ acting on the horizontal soil slice *i* by the FASS are given by the joint Eqs (17)–(19):


$$\{\begin{array}{l} p_{i}=\frac{D\left[q\left(b_{i}-b_{i-1}\right)+\left(G_{i}-G_{i-1}\right)+W_{spi}-c_{i}h_{i}\right]-B\left(c_{wi}h_{i}\cdot \cot\theta +c_{i}h_{i}\cdot \cot\alpha_{i}\right)}{BC}\\ \sigma_{pi}=\frac{D\left[q\left(b_{i}-b_{i-1}\right)+\left(G_{i}-G_{i-1}\right)+W_{spi}-c_{i}h_{i}\right]-B\left(c_{wi}h_{i}\cdot \cot\theta +c_{i}h_{i}\cdot \cot\alpha_{i}\right)}{BC\cdot h_{i}}\cdot \sin\theta \end{array}$$ (20)

The analytical expressions for the shear force $T_{pi}$ and the shear stress $\tau_{pi}$ acting on the horizontal soil slice *i* by the FASS are:


$$\{\begin{array}{l} T_{pi}=\left\{\frac{D\left[q\left(b_{i}-b_{i-1}\right)+\left(G_{i}-G_{i-1}\right)+W_{spi}-c_{i}h_{i}\right]-B\left(c_{wi}h_{i}\cdot \cot\theta +c_{i}h_{i}\cdot \cot\alpha_{i}\right)}{BC}\right\}\cdot \tan\delta_{i}+c_{wi}\cdot \frac{h_{i}}{\sin\theta }\\ \tau_{pi}=\left\{\frac{D\left[q\left(b_{i}-b_{i-1}\right)+\left(G_{i}-G_{i-1}\right)+W_{spi}-c_{i}h_{i}\right]-B\left(c_{wi}h_{i}\cdot \cot\theta +c_{i}h_{i}\cdot \cot\alpha_{i}\right)}{BC}\right\}\cdot \frac{\tan\delta_{i}\cdot \sin\theta }{h_{i}}+c_{wi} \end{array}$$ (21)

The analytical expressions for the vertical force $N_{i}$ and the vertical force strength $\sigma_{Ni}$ acting on the horizontal soil slice *i* by the sliding surface are:


$$\{\begin{array}{l} N_{i}=\frac{q\left(b_{i}-b_{i-1}\right)+\left(G_{i}-G_{i-1}\right)+W_{spi}-c_{i}h_{i}}{B}\\ \sigma_{Ni}=\frac{q\left(b_{i}-b_{i-1}\right)+\left(G_{i}-G_{i-1}\right)+W_{spi}-c_{i}h_{i}}{B\cdot h_{i}}\cdot \sin\alpha_{i} \end{array}$$ (22)

The analytical expressions for the shear force $T_{Ni}$ and the shear stress $\tau_{Ni}$ acting on the horizontal soil slice *i* by the sliding surface are:


$$\{\begin{array}{l} T_{Ni}=\left[\frac{q\left(b_{i}-b_{i-1}\right)+\left(G_{i}-G_{i-1}\right)+W_{spi}-c_{i}h_{i}}{B}\right]\cdot \tan\varphi_{i}+c_{i}\cdot \frac{h_{i}}{\sin\alpha_{i}}\\ \tau_{Ni}=\left[\frac{q\left(b_{i}-b_{i-1}\right)+\left(G_{i}-G_{i-1}\right)+W_{spi}-c_{i}h_{i}}{B}\right]\cdot \frac{\tan\varphi_{i}\cdot \sin\theta }{h_{i}}+c_{i} \end{array}$$ (23)

According to the principle of action force and reaction force, it can be obtained that the lateral earth pressure $e_{ai}$ perpendicular to the slope surface and the vertical force $p_{i}$ acting on the horizontal soil slice *i* by the FASS are equal in size and opposite in direction. Thus, the analytical expressions for the lateral earth pressure $e_{ai}$ and the lateral earth pressure strength $\sigma_{eai}$ perpendicular to the slope are:


$$\{\begin{array}{l} e_{ai}=\frac{D\left[q\left(b_{i}-b_{i-1}\right)+\left(G_{i}-G_{i-1}\right)+W_{spi}-c_{i}h_{i}\right]-B\left(c_{wi}h_{i}\cdot \cot\theta +c_{i}h_{i}\cdot \cot\alpha_{i}\right)}{BC}\\ \sigma_{eai}=\frac{D\left[q\left(b_{i}-b_{i-1}\right)+\left(G_{i}-G_{i-1}\right)+W_{spi}-c_{i}h_{i}\right]-B\left(c_{wi}h_{i}\cdot \cot\theta +c_{i}h_{i}\cdot \cot\alpha_{i}\right)}{BC\cdot h_{i}}\cdot \sin\theta \end{array}$$ (24)

The lateral earth pressure $e_{ai}$ perpendicular to the slope surface calculated for different horizontal soil slices *i* is summed to obtain the combined force $E_{a}$ of lateral soil pressures of the slope reinforced by the FASS, as shown in the following equation:


$$E_{\text{a}}=\sum e_{ai}=\sum \frac{D\left[q\left(b_{i}-b_{i-1}\right)+\left(G_{i}-G_{i-1}\right)+W_{spi}-c_{i}h_{i}\right]-B\left(c_{wi}h_{i}\cdot \cot\theta +c_{i}h_{i}\cdot \cot\alpha_{i}\right)}{BC}$$ (25)

## 4. Method validation

In order to verify the reasonableness and accuracy of the improved horizontal slice method for slope reinforced by the FASS proposed in this paper, the conventional algorithm for calculating lateral earth pressure on slopes is used in this section for comparison and verification with the algorithm in this paper.

**Fig 4:**
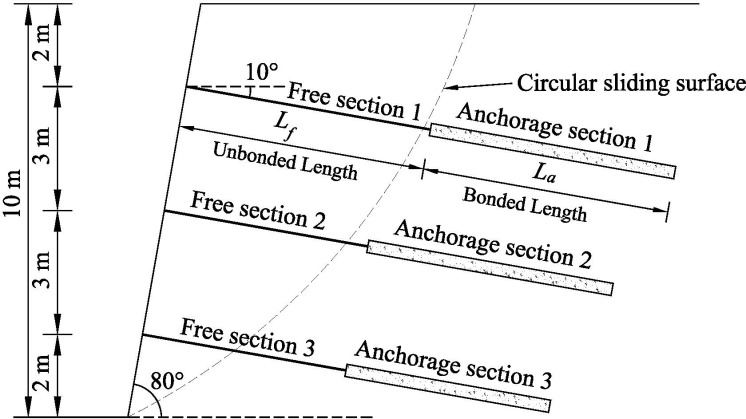
The diagram of slope reinforced by the FASS.

For the current slope angle is less than 90 ° and does not meet the Rankine earth pressure and Coulomb earth pressure assumption conditions of the lateral earth pressure calculation of the slope, scholars mostly take the overall sliding wedge method to calculate the combined force of lateral earth pressures on slopes, and based on the combined force of the design and analysis of slopes as well as the distribution of the lateral earth pressure strength study. Therefore, in order to verify the reasonableness and accuracy of the calculation method of lateral earth pressure based on the improved horizontal slice method for slope reinforced by the FASS proposed in this paper, this section takes the homogeneous single-stage of slope reinforced by the FASS as the research object (shown in Fig 4), and uses the calculation method of this paper and the overall sliding wedge method to compare and analyse the combined force of the lateral earth pressures and the lateral earth pressure strength on slopes, respectively.

### 4.1. Overview of calculation example

The slope has a height of 10 m, a gradient of 80°, and a uniform load of 20 kN/m^2^ at the top of the slope. Three anchors are provided at the positions of 2 m, 5 m, and 8 m of the slope, and the inclination angle of the anchors is 10°, and the specific parameters of the anchors are shown in Table 1. The slope consists of uniformly distributed silty clay with soil parameters shown in Table 2.

**Table 1:** The parameter of anchor.

Number	Diameter of steel bar	Anchor length	Unbonded length	Bonded length	Diameter of anchorage section
**1**	25 mm	12 m	6 m	6 m	150 mm
**2**	25 mm	11 m	5 m	6 m	150 mm
**3**	25 mm	10 m	5 m	5 m	150 mm

**Table 2:** The parameter of soil.

Soil	Weight	Cohesion	Internal friction angle	Poisson’s ratio	Elastic modulus
**Silty Clay**	16.4 kN/m^3^	18 kPa	25°	0.3	10.5 MPa

### 4.2. The calculation model of overall sliding wedge method

**Fig 5:**
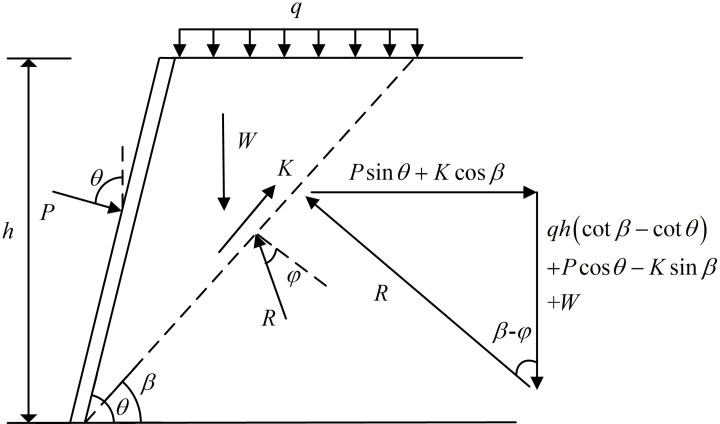
The calculation model of overall sliding wedge method.

The lateral earth pressure calculation model for the overall sliding wedge method is shown in Fig 5. When the sliding wedge is in limiting equilibrium, the force analysis is carried out with the sliding wedge as the object of study. This method is more accurate in calculating the combined force of lateral earth pressures on the slope. The sliding wedge is subjected to the uniform load *q* at the top of the slope, its own gravity *W*, the cohesive force *K* of the stable soil in the lower part of the sliding surface acting on the sliding wedge, the resisting force *P* of the FASS acting on the sliding wedge, and the counter-force *R* of the stable soil acting on the sliding wedge (*R* is the combined force of the normal support force of the stable soil acting on the sliding wedge and the friction force upwards along the sliding surface.). The sliding wedge is in equilibrium under the above external forces.

The self-weight *W* of the sliding wedge per linear metre of soil is:


$$W=\frac{1}{2}\gamma h^{2}\left(\cot\beta -\cot\theta \right)$$ (26)

The cohesive force *K* acting on the sliding wedge by the stable soil below the sliding surface is:


$$K=\frac{h}{\sin\beta }c$$ (27)

According to the principle of force interaction, the analytical expression for the combined force of lateral earth pressures acting on the FASS by the sliding wedge is given by:


$$E_{a}=P=\frac{\tan\left(\beta -\varphi \right)\left[\left(\frac{1}{2}\gamma h^{2}+qh\right)\left(\cot\beta -\cot\theta \right)-hc\right]-hc\cot\beta }{\sin\theta -\tan\left(\beta -\varphi \right)\cos\theta }$$ (28)

By taking the derivative of *h* in Eq (28), the analytical expression for the lateral earth pressure strength on slopes can be obtained as follows:


$$e_{a}=\frac{\text{d}P}{\text{d}h}=\frac{\tan\left(\beta -\varphi \right)\left[\left(\gamma h+q\right)\left(\cot\beta -\cot\theta \right)-c\right]-c\cot\beta }{\sin\theta -\tan\left(\beta -\varphi \right)\cos\theta }$$ (29)

### 4.3. Comparative analysis of calculation results

In calculating lateral earth pressure, the calculation method of this paper and the overall sliding wedge method need to determine the location of the most dangerous sliding surface, and then the corresponding parameter values are substituted into the relevant formulae for the solution of lateral earth pressure.

#### 4.3.1. Determination of the sliding surface of slope.

**Fig 6:**
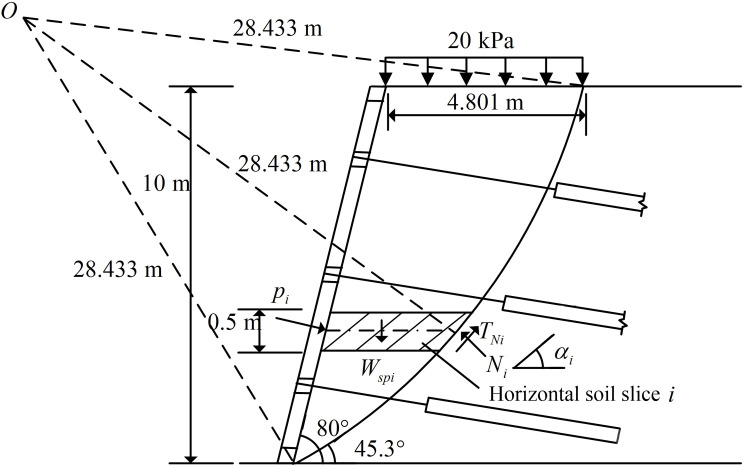
The diagram of most dangerous potential circular sliding surface.

For the improved horizontal slice method for slope reinforced by the FASS proposed in this paper, it is assumed that the most dangerous potential sliding surface is a circular sliding surface, and the exact location is determined by Geo-Studio finite element analysis software. The location of the most dangerous potential sliding surface is shown in Fig 6, which has a radius of 28.433 m of the circular sliding surface.

**Fig 7:**
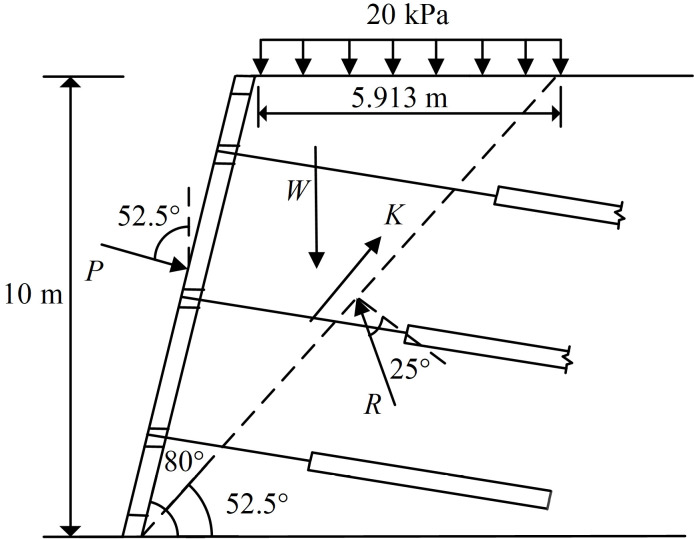
The diagram of sliding surface of the overall sliding wedge method.


For the overall sliding wedge method, the most dangerous potential sliding surface is solved by the classical formula for the potential angle of rupture of the slope (when $\text{d}E_{a}/\text{d}\beta =0$, calculate the potential fracture angle $\beta_{f}$ of the slope). The formula for calculating the potential angle of rupture $\beta_{f}$ of the slope is shown in Eq (30). The potential angle of rupture of the slope for this example is calculated to be 52.5°, as shown in Fig 7.

$$\beta_{f}=\frac{\theta +\varphi }{2}$$ (30)

#### 4.3.2. Calculation of lateral earth pressure on the slope reinforced by the FASS.

Based on the calculation formula of earth pressure proposed in this paper and the calculation formula of earth pressure proposed by the integral sliding wedge method, and combined with the determination of the potential sliding surface by different calculation methods, the calculated values of lateral earth pressure of the slope reinforced by the FASS are obtained by the two calculation methods respectively.

**(1) Comparison of the combined force of lateral earth pressures on the slope reinforced by the FASS:** When using the calculation method in this paper, the friction angle $\delta_{i}$ and cohesion $c_{wi}$ between the wall and soil at the location of the horizontal soil slice *i* involved in the formula are generally measured values, which can be selected according to the following formula in the absence of relevant information:


$$\{\begin{array}{l} \delta_{i}=\frac{2}{3}\varphi_{i}\\ c_{wi}=\frac{2}{3}c_{i} \end{array}$$ (31)

where $\varphi_{i}$ denotes the angle of internal friction of the soil at the location of the horizontal soil slice *i* corresponding to the sliding surface; $c_{i}$ denotes the cohesion of the soil at the location of the horizontal soil slice *i* corresponding to the sliding surface.

By substituting the relevant parameters of this example into Eq (25) and Eq (28), the combined forces of lateral earth pressures of the slope reinforced by the FASS are obtained by the two calculation methods respectively, as shown in Table 3 below. Because the soil is silty clay (cohesion exists), the lateral earth pressure of the soil slice near the foot of the slope is negative when using the calculation method in this paper (the negative value is small). However, the soil at the foot of the slope is not subject to tension in the actual project, so it is assumed that the negative lateral earth pressure at the foot of the slope is not considered when calculating the combined force of lateral earth pressures. The negative lateral earth pressure at the foot of the slope is corrected for a distribution of lateral earth pressures that converge infinitely to 0.

**Table 3:** Comparison of the combined force of lateral earth pressures.

Calculation method	Calculation method in this paper	Overall sliding wedge method
**Combined force of lateral earth pressures**	83.6 kN	91.7 kN

As seen from Table 3, the combined force of lateral earth pressures obtained by the calculation method of this paper is smaller than that of the overall sliding wedge method, but the difference between the two calculation methods is not large. The difference between the two methods is 8.1 kN (accounting for 8.8% of the combined force value of lateral earth pressures obtained by the overall sliding wedge method). The main reason for analysing the results of the above comparisons is that the calculation method in this paper mainly takes into account the following factors compared with the overall sliding wedge method: (a) The inter-slice force of the soil slice is considered; (b) The action of the frame columns in the FASS on the slope is considered; (c) The circular sliding surface more in line with the reality of the slope is used.

**Fig 8:**
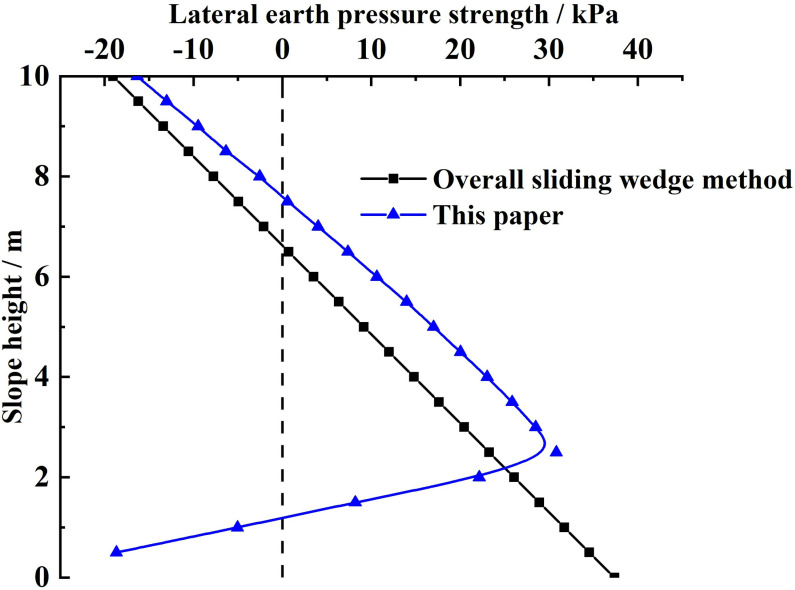
Comparison of lateral earth pressure strength before correction.

**Fig 9:**
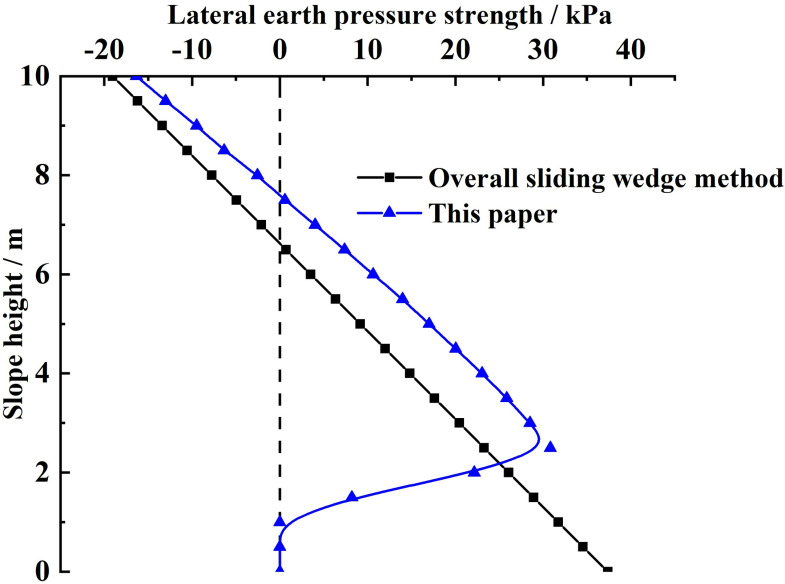
Comparison of lateral earth pressure strength after correction.

**(2) Comparison of the distribution of lateral earth pressure strengths on the slope reinforced by the FASS:** By substituting the relevant parameters of this example into Eq (24) and Eq (29), the lateral earth pressure strengths of the slope reinforced by the FASS along the slope height are obtained by the two calculation methods respectively, as shown in Fig 8. Considering that the soil at the foot of the slope is not subject to tension, Fig 8 is corrected, and the corrected negative lateral earth pressure strength at the foot of the slope is 0. The corrected lateral earth strength distribution is shown in Fig 9.

From Figs 8 and 9, it can be seen that there is a negative case (within a specific range from the top of the slope) for the lateral earth pressure strength obtained by both calculation methods because the study object is the silty clay. However, the calculation method in this paper is not the same as the distribution of lateral earth pressure strength along the slope height obtained by the overall sliding wedge method. For the overall sliding wedge method, the lateral earth pressure strength increases gradually and linearly from a negative value to a positive maximum value as the slope height decreases. The lateral earth pressure strength obtained by the calculation method in this paper shows a nonlinear distribution of small middle at both ends along the slope height direction (the maximum value appears in the middle of the slope to the foot of the slope), i.e., it is firstly increased nonlinearly from the negative value to the positive maximum value, and then decreased nonlinearly from the maximum value.

Combined with the existing experimental research on the distribution of lateral earth pressure strength of slopes [40–42], it can be seen that the distribution of lateral earth pressure strength obtained by the calculation method of this paper, as well as the location of the maximum value of the actual lateral earth pressure strength of slopes in line with the variety rule of the actual lateral earth pressure strength.

The reason why the distribution of earth pressure strength obtained by the calculation method in this paper is different from the overall sliding wedge method is:

(a) The overall sliding wedge method in the calculation of soil pressure strength is only the derivative of the combined force, did not take into account the range of influence of external loads on the sliding soil, resulting in external loads on the sliding soil have the same effect. In contrast, the calculation method in this paper uses the horizontal slice method to analyze the force on each soil slice in the sliding zone, which takes into account the range of influence of external loads in the sliding soil.
(b) The overall sliding wedge method uses the rupture angle to determine the potential sliding surface, and the rupture angle on the potential sliding surface is the same, resulting in a linear distribution of earth pressure strength at the different slope heights. In contrast, the calculation method of this paper adopts the circular sliding surface method, which fully considers the nonlinear damage characteristics of sliding soil with different slope heights, and provides the necessary conditions for the nonlinear distribution of earth pressure strength.
(c) In practice situations, the earth pressures strength at the different slope heights is affected by the combined action of the upper and lower soils. The overall sliding wedge method does not take into account the effect of the upper and lower soils on earth pressure strength. In contrast, the calculation method in this paper obtains the earth pressure strength considering the action of upper and lower soils by analyzing the force of horizontal soil slice force at the different slope heights.

Therefore, the distribution law of earth pressure strength obtained by the calculation method in this paper satisfies the existing experimental study on the distribution of earth pressure strength. That is, the earth pressure strength shows a nonlinear distribution of small middle at both ends along the slope height direction. And the overall sliding wedge shows a linear distribution of monotonically increasing.

## 5. Results and discussion

In order to further understand the variety rule of the relevant parameters calculated by the calculation method of this paper, this section will carry out the study and analysis of the variety rule of the relevant parameters under the action of different slope-top uniform loads. The main areas include the following four: (1) the combined force of lateral earth pressures and the lateral earth pressure strength perpendicular to the slope; (2) the combined force of shear forces and the shear stress acting on the sliding soils by the FASS; (3) the combined force of the vertical forces and the vertical force strength acting on the sliding soils by the sliding surface; (4) the combined force of shear forces and the shear stress acting on the sliding soils by the sliding surface.

Before starting the research and analysis of the above four aspects, the following assumptions are made in this section: (1) the potential sliding surface of the slope does not change under different slope-top uniform loads; (2) when calculating the combined force of each parameter, only the positive values are considered ignoring the negative values. The values taken for different slope-top uniform loads are shown in Table 4.

**Table 4:** Different slope-top uniform loads.

Type of load	Slope-top uniform load						
**Value**	0 kPa	20 kPa	40 kPa	60 kPa	80 kPa	100 kPa	120 kPa

### 5.1. Analysis of the combined force of lateral earth pressures and the lateral earth pressure strength under different slope-top uniform loads

**Fig 10:**
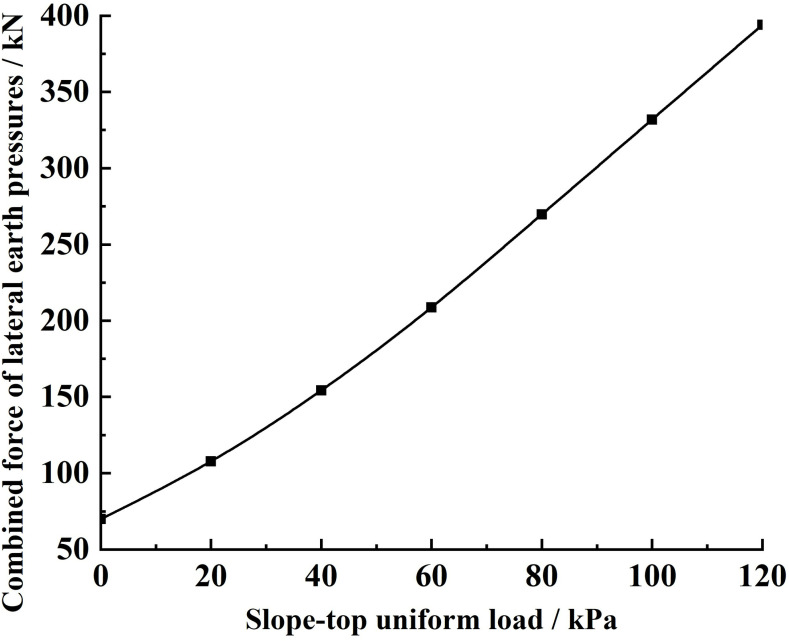
Variation of lateral earth pressure under different slope-top uniform loads: combined force of lateral earth pressures.

**Fig 11:**
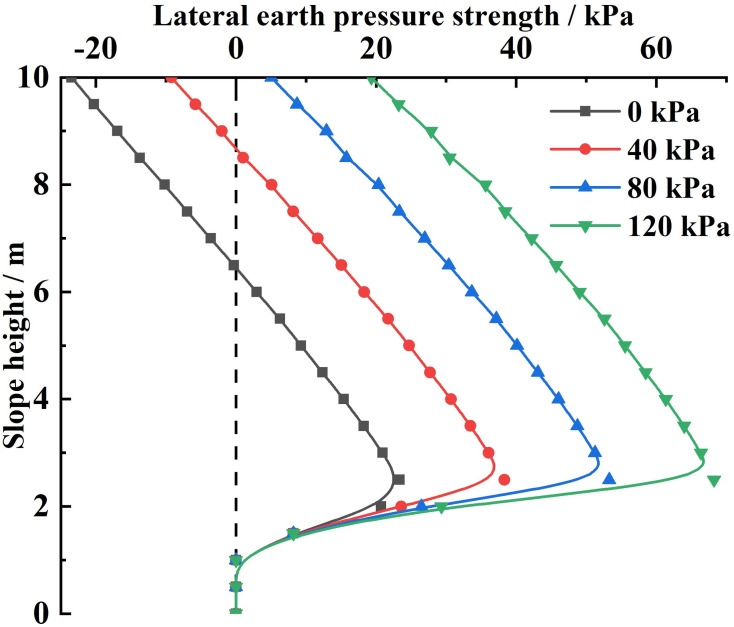
Variation of lateral earth pressure under different slope-top uniform loads: lateral earth pressure strength.

Substituting the relevant parameters and the different slope-top uniform loads into Eq (24), the variety rule of the combined force of lateral earth pressures and the lateral earth pressure strength under different slope-top uniform loads is obtained, as shown in Figs 10 and 11.

As shown in Fig 10, the combined force of lateral earth pressures under different slope-top uniform loads shows the increasing rule of variety with the increase of slope-top uniform load. The magnitude of the slope-top uniform load affects the rate of increase of the combined force of lateral earth pressures, with a slower rate of increase for slope-top uniform loads less than 60 kPa and a faster rate of increase for slope-top uniform loads more significant than 60 kPa, but the difference between the two rates of change is not significant. Therefore, it can be assumed that the combined force of lateral earth pressures on the slope reinforced by the FASS exhibits an approximately linear increase in the variety rule with the increase of the slope-top uniform load.

As can be seen from Fig 11, the lateral earth pressure strength of the slope reinforced by the FASS under different slope-top uniform loads shows a distribution trend of small at both ends and large in the middle along the height direction of the slope, with its maximum value occurring at about 1/3 of the position from the bottom of the slope. The variety rule indicates that for the same slope reinforced by the FASS, the location of the maximum value of lateral earth pressure strength does not vary with the change of the slope-top uniform load.

In addition, according to Fig 11, it can also be obtained that the negative value at the top of the slope caused by soil cohesion will gradually increase to the positive value with the increasing slope-top uniform load. At the same time, the value of the slope’s maximum lateral earth pressure strength under different slope-top uniform loads also increases with the increasing slope-top uniform loads. For the distribution of lateral earth pressure strength, with increasing slope-top uniform loads, the distribution of lateral earth pressure strength will gradually shift positively towards the coordinate axis, which leads to a sharp increase in the rate of decrease of the maximum lateral earth pressure strength towards the foot of the slope.

Analyze the location of the maximum value of earth pressure strength. The horizontal soil slice at this location is located where the vertical line at the top of the slope meets the circular sliping surface and has the following characteristics:

(1) In terms of forces, the horizontal soil slice at this location is the last horizontal soil slice directly affected by the vertical loads. All horizontal soil slices after this location will not be directly affected by vertical loads at the top of the slope, and the loads will only be taken into account through the interaction between horizontal soil slices.
(2) In geometric terms, the upper top surfaces of the vertical soil slices corresponding to all horizontal soil slices after this location are located on the slope surface and not at the top of the slope. And the height of the vertical soil slice decreases sharply with decreasing distance from the bottom of the slope.

### 5.2. Analysis of the combined force of shear forces and the shear stress acting on the sliding soils by the FASS under different slope-top uniform loads

**Fig 12:**
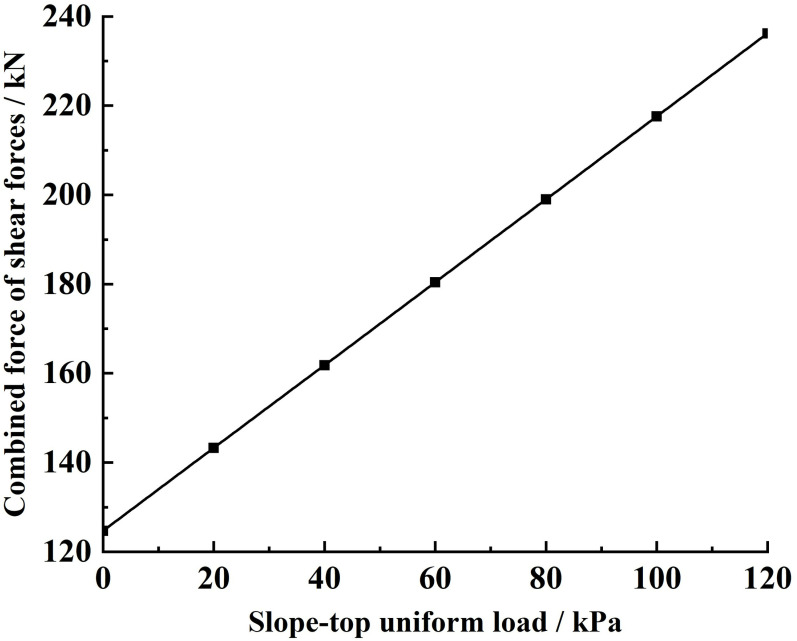
Variation of shear force acting on the sliding soils by the FASS under different slope-top uniform loads: combined force of shear forces.

**Fig 13:**
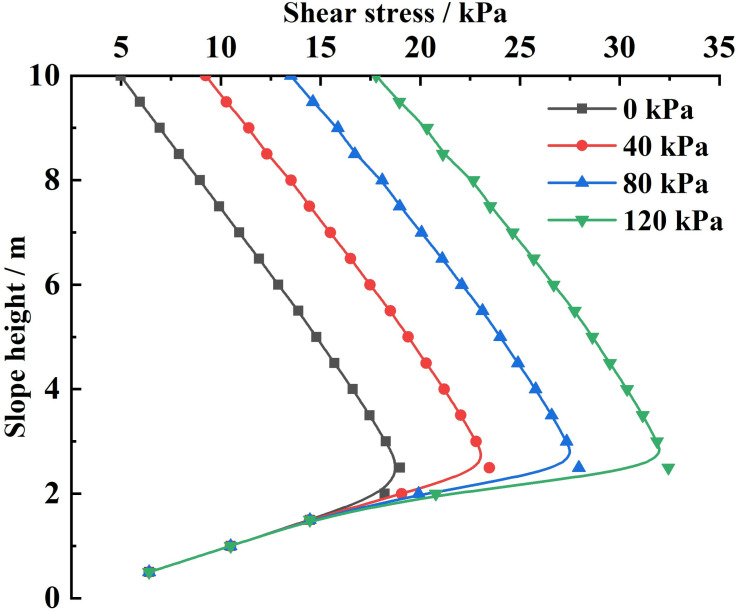
Variation of shear force acting on the sliding soils by the FASS under different slope-top uniform loads: shear stress.

Substituting the relevant parameters and the different slope-top uniform loads into Eq (21), the variety rule of the combined force of shear forces and the shear stress acting on the sliding soils by the FASS under different slope-top uniform loads is obtained, as shown in Figs 12 and 13.

From Fig 12, it can be seen that the combined force of shear forces acting on the sliding soils by the FASS under different slope-top uniform loads shows an approximately linear increase in the distribution law with the increase of the slope-top uniform load; and in terms of numerical magnitude, the combined force of shear forces acting on the sliding soils by the FASS is greater than the combined force of the lateral earth pressures generated by the slope under the same uniform load at the top of the slope. The above variety rule shows that: the shear force acting on the sliding soils by the FASS has a specific role in resisting the sliding of the slope along the potential sliding surface, and the shear force acting on the sliding soils by the FASS should not be neglected in the force and stability analysis of the slope reinforced by the FASS.

As can be seen from Fig 13, the shear stress acting on the sliding soils by the FASS under different slope-top uniform loads shows a distribution trend of small at both ends and large in the middle along the height direction of the slope, with its maximum value occurring at about 1/3 of the position from the bottom of the slope (the location of the maximum value does not vary with the slope-top uniform loads), and the shear stresses are all positive along the slope height direction (parallel to the slope surface upwards). At the same time, with the continuous increase of the slope-top uniform load, the distribution of the shear stress acting on the sliding soils by the FASS will be gradually shifted to the positive direction of the coordinate axis, the maximum shear stress value will gradually increase, and the reduction rate of the shear stress from the maximum to the minimum at the foot of the slope will increase sharply.

Analysis of the above variety rule can be found: (1) the variety rule of the shear stress acting on the sliding soils by the FASS is the same as the variety rule of the lateral earth pressure strength, indicating that it is closely related to the range of horizontal soil slices affected by the slope-top uniform load; (2) The shear stress generated by the FASS is distributed along the slope height direction and its value is significant, so the influence of the shear stress acting on the sliding soils by the FASS should be fully considered in the design and analysis of the slope reinforced by the FASS.

### 5.3. Analysis of the combined force of the vertical forces and the vertical force strength acting on the sliding soils by the sliding surface under different slope-top uniform loads

**Fig 14:**
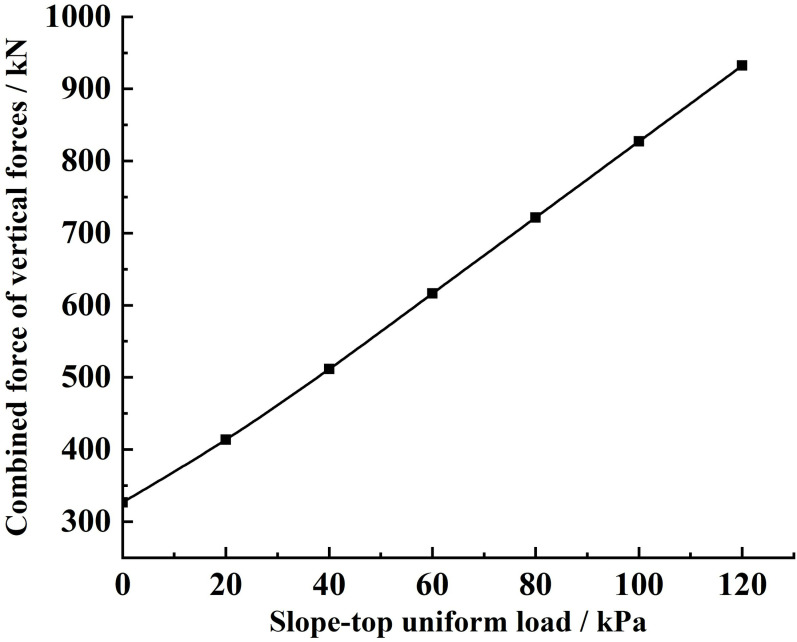
Variation of vertical force acting on the sliding soils by the sliding surface under different slope-top uniform loads: combined force of vertical forces.

**Fig 15:**
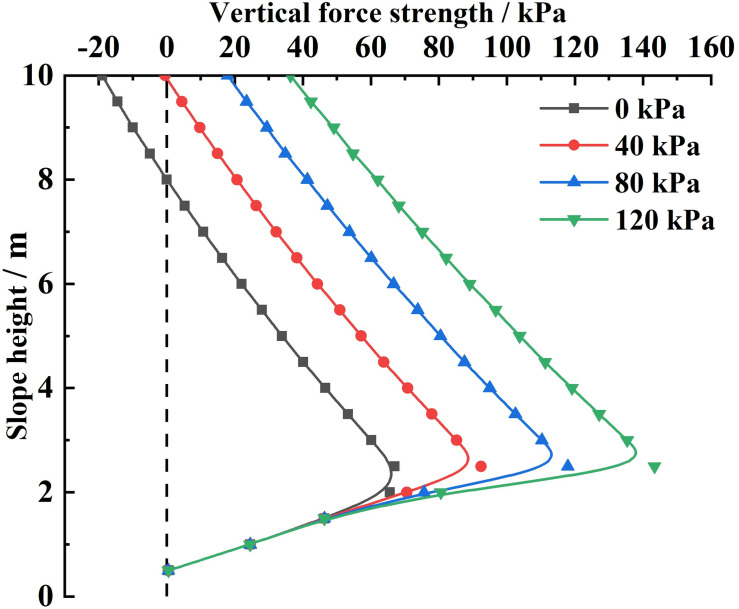
Variation of vertical force acting on the sliding soils by the sliding surface under different slope-top uniform loads: vertical force strength.

Substituting the relevant parameters and the different slope-top uniform loads into Eq (22), the variety rule of the combined force of the vertical forces and the vertical force strength acting on the sliding soils by the sliding surface under different slope-top uniform loads is obtained, as shown in Figs 14 and 15.

From Fig 14, it can be seen that the combined force of the vertical forces acting on the sliding soils by the sliding surface under different slope-top uniform loads shows an approximately linear increase in the distribution law with the increase of the slope-top uniform load; the combined force of the vertical forces in Fig 14 is about three times the combined force of lateral earth pressures under the same slope-top uniform load.

According to the above variety, the slope-top uniform load significantly influences on the vertical force acting on the sliding soils by the sliding surface. That is, when the slope is subjected to a large slope-top uniform load, the stabilising soil will generate a large vertical force to prevent the sliding soil from sliding.

As can be seen from Fig 15, the vertical force strength acting on the sliding soils by the sliding surface under different slope-top uniform loads shows a distribution trend of small at both ends and large in the middle along the height direction of the slope, with its maximum value occurring at about 1/3 of the position from the bottom of the slope (the location of the maximum value does not vary with the slope-top uniform loads). This trend is the same as the shear stress acting on the sliding soils by the FASS and the lateral earth pressure strength. However, the vertical force strength acting on the sliding soils by the sliding surface is much greater than the earth pressure strength and the shear stress acting on the sliding soils by the FASS under the same load.

Analysing the above variety rule, it can be found that the variety rule of the vertical force strength acting on the sliding soils by the sliding surface is related to the scope of influence of the slope-top uniform load. That is, outside the range of influence of the slope-top uniform load, the vertical force strength acting on the sliding soils by the sliding surface will be significantly reduced.

### 5.4. Analysis of the combined force of shear forces and the shear stress acting on the sliding soils by the sliding surface under different slope-top uniform loads

**Fig 16:**
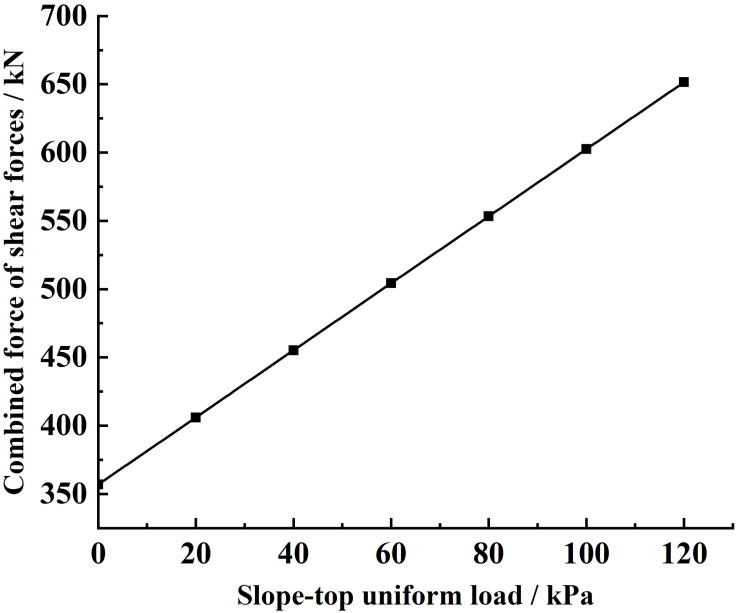
Variation of shear force acting on the sliding soils by the sliding surface under different slope-top uniform loads: combined force of shear forces.

**Fig 17:**
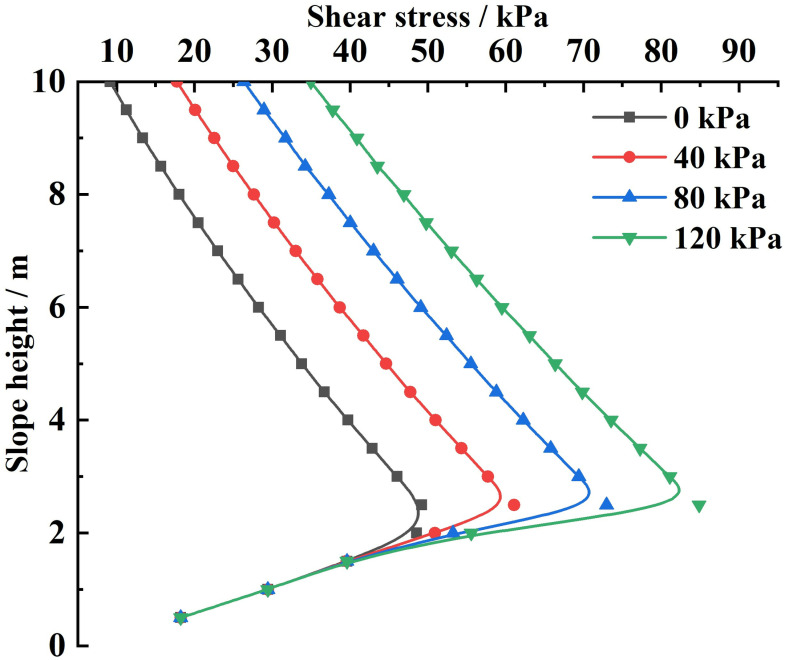
Variation of shear force acting on the sliding soils by the sliding surface under different slope-top uniform loads: shear stress.

Substituting the relevant parameters and the different slope-top uniform loads into Eq (23), the variety rule of the combined force of shear forces and the shear stress acting on the sliding soils by the sliding surface under different slope-top uniform loads is obtained, as shown in Figs 16 and 17.

From Fig 16, it can be seen that the combined force of shear forces acting on the sliding soils by the sliding surface under different slope-top uniform loads shows an approximately linear increase in the distribution law with the increase of the slope-top uniform load. The trend is the same as that of the combined force of lateral earth pressures, the combined force of shear forces and the shear stress acting on the sliding soils by the FASS, and the combined force of the vertical forces acting on the sliding soils by the sliding surface. In addition, the combined force of shear forces acting on the sliding soils by the sliding surface under the same slope-top uniform load is much larger than the combined force of shear forces acting on the sliding soils by the FASS, which indicates that the combined force of shear forces acting on the sliding soils by the sliding surface plays an important role in resisting the sliding soil from sliding.

As can be seen from Fig 17, the shear stress acting on the sliding soils by the sliding surface under different slope-top uniform loads shows a distribution trend of small at both ends and large in the middle along the height direction of the slope, with its maximum value occurring at about 1/3 of the position from the bottom of the slope (the location of the maximum value does not vary with the slope-top uniform loads). The variety rule is the same as that of the parameter above. Meanwhile, since the shear stress acting on the sliding soils by the sliding surface acts as the main source of resistance to slope sliding, its value is positive under different slope-top uniform loads.

Analysing the specific value of the shear stress acting on the sliding soils by the sliding surface, it can be found that the value is much larger than the shear stress acting on the sliding soils by the FASS, and this result further indicates that the stabilising soil plays a crucial role in resisting the sliding soil from sliding.

### 5.5. Engineering application analysis

According to the above research results, combined with the force analysis of slope reinforced by FASS in practical engineering, it is believed that the research results will provide theoretical basis and reference for practical engineering application in the following aspects:

(1) The method of force analysis and force calculation of slope reinforced by FASS proposed in this paper is in line with the forces of actual slope reinforced by FASS. This method will provide data support that is close to the actual situation of the slope for the calculation of the stability of slope reinforced by FASS. And, this method is also convenient for calculating the actual stability of slope reinforced by FASS.
(2) The method in this paper improves the accuracy of force calculation and analysis of slope reinforced by FASS. This method can propose a more economical and safe design of FASS for the actual force and distribution of slope reinforced by FASS.
(3) According to the distribution of the actual force of the slope reinforced by FASS obtained by the method of this paper, the most dangerous location of the slope can be determined and provide a reference for the arrangement of the monitoring point of the slope reinforced by FASS.

## 6. Conclusions

In this paper, based on the principle that the vertical slice method and the horizontal slice method share the same sliding surface, an improved horizontal slice method for slope reinforced by the FASS considering the influence of multiple factors is proposed. Moreover, by comparing and analyzing specific examples of calculations, the reasonableness and accuracy of the calculation method of this paper are verified, and the variety rule of force on the slope reinforced by FASS under different slope-top uniform loads is obtained. The following conclusions can be drawn from the above studies and analyses:

(1) Compared with the overall sliding wedge method, the improved horizontal slice method for slope reinforced by the FASS proposed in this paper considers the influence of the inter-strip force between soil slices and the force of the FASS on the slope face. This makes the combined force of lateral earth pressures on slopes calculated in this paper is smaller than that of the overall sliding wedge method, but the difference between the two is not significant (within 9% difference). At the same time, the lateral earth pressure strength of the slope obtained by the calculation method of this paper shows a distribution trend of small at both ends and large in the middle along the height direction of the slope, with its maximum value occurring at about 1/3 of the position from the bottom of the slope. The variety rule aligns with the existing relevant experimental research, which verifies the reasonableness and accuracy of the improved horizontal slice method for slope reinforced by the FASS.
(2) For the overall forces on the slope, the combined force of lateral earth pressures, the combined force of shear forces acting on the sliding soils by the FASS, and the combined force of the vertical forces and the combined force of shear forces acting on the sliding soils by the sliding surface obtained by the improved horizontal slice method for slope reinforced by the FASS under different slope-top uniform loads all show an approximately linear distribution with the increase of the slope-top uniform load.
(3) For the distribution of force along the slope height direction, the lateral earth pressure strength, the shear stress acting on the sliding soils by the FASS, and the vertical force strength and the shear stress acting on the sliding soils by the sliding surface obtained by the improved horizontal slice method for slope reinforced by the FASS under different slope-top uniform loads all show the distribution trend of small at both ends and large in the middle along the height direction of the slope. The maximum values of the above parameters all occur in the middle and lower part of the slope (about 1/3 of the position from the bottom of the slope), and the trend does not vary with the slope-top uniform load.
(4) The force analysis of horizontal soil slices using the improved horizontal slice method for slope reinforced by the FASS reveals that the distribution trend of the relevant force along the slope height direction is closely related to the range of influence of the slope-top uniform load on the soil. That is, outside the range of influence of the slope-top uniform load, the forces associated with the slope will be significantly reduced, and the maximum of the forces associated with the slope will occur at the demarcation location.
(5) The FASS will exert shear force on the sliding soil (its value is about 1/3 of the shear force of the stabilized soil on the sliding soil). The shear force of the FASS on the slope should not be neglected in the calculation of the force and stability of the slope reinforced by FASS.
(6) The study in this paper is on the force analysis and calculation of slope reinforced by FASS under static conditions. However, the application environment of slope reinforced by FASS is complex in the actual engineering. Therefore, it is also necessary to study the force analysis and calculation of slope reinforced by FASS under different application scenarios such as rainfall and earthquake. And further consideration needs to be given in the subsequent study to the effect of the length of the anchor free section and the anchor anchorage section on the calculation results of this paper in the special case where the sliping surface does not pass through the anchor free section.

## Supporting information

S1 Data Figure data.
(XLSX)
